# Identification of genetic alterations in pancreatic cancer by the combined use of tissue microdissection and array-based comparative genomic hybridisation

**DOI:** 10.1038/sj.bjc.6603563

**Published:** 2007-01-23

**Authors:** T Harada, P Baril, R Gangeswaran, G Kelly, C Chelala, V Bhakta, K Caulee, P C Mahon, N R Lemoine

**Affiliations:** 1 Centre for Molecular Oncology, Institute of Cancer, Barts and The London School of Medicine and Dentistry, Queen Mary University of London, Charterhouse Square, London EC1M 6BQ, UK; 2 Cancer Research UK Clinical Centre, Barts and The London School of Medicine and Dentistry, Queen Mary University of London, Charterhouse Square, London EC1M 6BQ, UK; 3 Cancer Research UK, Bioinformatics and Biostatistics Service, Lincoln's Inn Fields, London WC2A 3PX, UK

**Keywords:** pancreatic cancer, tissue microdissection, array CGH, *SEC11L3*

## Abstract

Pancreatic ductal adenocarcinoma (PDAC) is characterised pathologically by a marked desmoplastic stromal reaction that significantly reduces the sensitivity and specificity of cytogenetic analysis. To identify genetic alterations that reflect the characteristics of the tumour *in vivo*, we screened a total of 23 microdissected PDAC tissue samples using array-based comparative genomic hybridisation (array CGH) with 1 Mb resolution. Highly stringent statistical analysis enabled us to define the regions of nonrandom genomic changes. We detected a total of 41 contiguous regions (>3.0 Mb) of copy number changes, such as a genetic gain at 7p22.2–p15.1 (26.0 Mb) and losses at 17p13.3–p11.2 (13.6 Mb), 18q21.2–q22.1 (12.0 Mb), 18q22.3–q23 (7.1 Mb) and 18q12.3–q21.2 (6.9 Mb). To validate our array CGH results, fluorescence *in situ* hybridisation was performed using four probes from those regions, showing that these genetic alterations were observed in 37–68% of a separate sample set of 19 PDAC cases. In particular, deletion of the *SEC11L3* gene (18q21.32) was detected at a very high frequency (13 out of 19 cases; 68%) and *in situ* RNA hybridisation for this gene demonstrated a significant correlation between deletion and expression levels. It was further confirmed by reverse transcription–PCR that *SEC11L3* mRNA was downregulated in 16 out of 16 PDAC tissues (100%). In conclusion, the combination of tissue microdissection and array CGH provided a valid data set that represents *in vivo* genetic changes in PDAC. Our results raise the possibility that the *SEC11L3* gene may play a role as a tumour suppressor in this disease.

Pancreatic ductal adenocarcinoma (PDAC) is the fourth leading cause of cancer-related death in Western countries. Although the prognosis of patients with many types of cancer has improved recently due to advances in diagnostic and therapeutic modalities, the outlook for patients with PDAC still remains dismal with a median survival of just 6 months from diagnosis and an overall 5-year survival rate of less than 5% (Warshaw and Fernandez-del Castillo, 1992; Murr *et al*, 1994; Jemal *et al*, 2005). Many previous studies have attempted to elucidate the molecular mechanisms underlying pancreatic tumorigenesis, but it is still not fully understood. Therefore, a better understanding of the genes involved in tumour growth and progression is necessary for the development of novel diagnostic and therapeutic strategies that could improve the outcome of this deadly disease.

Array-based comparative genomic hybridisation (array CGH) is a powerful technique that has been used to detect DNA copy number alterations across the entire genome of malignant tumours (Solinas-Toldo *et al*, 1997; Pinkel *et al*, 1998; Pollack *et al*, 1999; Albertson *et al*, 2000; Snijders *et al*, 2001; Fiegler *et al*, 2003). Compared to conventional CGH, the significantly improved resolution of array CGH permits highly accurate mapping of DNA copy number changes throughout the genome. In cancer, genomic alterations contribute to dysregulation of the expression levels of oncogenes and tumour suppressor genes, the accumulation of which is correlated with tumour progression (Ried *et al*, 1999). Therefore, array CGH provides a promising starting point for the identification of novel candidate genes affected by such genomic imbalances. Several array CGH investigations of PDAC have already been reported, but all these studies were performed on cell lines or whole tissue samples (Aguirre *et al*, 2004; Heidenblad *et al*, 2004; Holzmann *et al*, 2004; Mahlamaki *et al*, 2004; Bashyam *et al*, 2005; Gysin *et al*, 2005; Nowak *et al*, 2005). In cell lines, culture-induced genetic adaptation may be induced during the establishment of cell lines in *in vitro* conditions. On the other hand, PDAC tissues are characterised by a desmoplastic reaction, with neoplastic cells constituting only a small proportion of the tumour mass. Therefore, cytogenetic analysis using bulk tissue samples is invariably hampered by contamination with non-neoplastic cells.

The aim of this study is to identify novel genetic abnormalities that precisely reflect the characteristics of tumour cells *in vivo*. For this purpose, we applied array CGH to 23 microdissected PDAC tissue samples that consist of purified populations of cancer cells. Then, a subset of identified genetic alterations was evaluated in an independent sample set of 19 PDAC cases using fluorescence *in situ* hybridisation (FISH) analysis. Finally, *in situ* RNA hybridisation (ISH) and reverse transcription–PCR (RT–PCR) were performed to assess whether the identified genetic alteration could lead to significant change in transcript level of the gene in question.

## MATERIALS AND METHODS

### Tissue samples

A total of 23 fresh-frozen PDAC specimens were obtained surgically or at autopsy from Yamaguchi University School of Medicine, Japan, with appropriate ethical approval (Table 1). All the tissues were confirmed histologically by a pathologist. Tissue microdissection was performed manually to collect more than 90% of tumour cells in all the cases as described previously (Harada *et al*, 2002a). Briefly, only cancerous regions were microdissected using a sterile 26-gauge needle from several serial tissue sections (20 *μ*m thickness) under visualisation with an inverted microscope (Nikon 66906, Tokyo, Japan). DNA was extracted from at least 5000 tumour cells according to the standard protocol. Reference DNA was obtained from lymphocytes of both healthy male and female volunteers. In addition, another series of 19 formalin-fixed, paraffin-embedded tumour sections (4 *μ*m thickness) were provided from Yamaguchi University (*n*=10) and Tohoku University School of Medicine, Japan (*n*=9), for FISH and ISH analyses (Table 1). Owing to the limited accessibility of clinical specimens, the samples used in array CGH were not available for FISH and ISH. For RT–PCR, 16 fresh-frozen PDAC tissues and two normal pancreas tissues were obtained from the Human Biomaterials Resource Centre, Department of Histopathology, Charing Cross Hospital, London, UK, with full ethical approval of the host institution. The clinicopathological information was not available for these anonymous samples. Haematoxylin–eosin-stained slides were examined to ensure a content of 60–80% tumour cells before use and then, total RNA was extracted directly from homogenised tissues using TRIZOL (Invitrogen Ltd, Paisley, UK).

### CGH arrays and image acquisition

The whole-genome CGH arrays were produced at the Wellcome Trust Sanger Institute and consist of 3125 BAC/PAC clones that cover the entire human genome at 1 Mb resolution (Fiegler *et al*, 2003). All the clone details are available on the Ensembl genome browser (v39, June 2006; http://www.ensembl.org/Homo_sapiens/index.html).

Array CGH was performed as described previously, with minor modifications (Fiegler *et al*, 2003; Hurst *et al*, 2004). Briefly, tumour and reference DNAs (300 ng) were labelled with Cy5-dCTP and Cy3-dCTP, respectively. Hybridisation was carried out at 37°C for 36 h in a hybridisation chamber (GeneMachines, San Carlos, CA, USA). After washing the slides, fluorescence intensities were measured on an Axon 4000B scanner (Axon Instruments Inc., Burlingame, CA, USA) and the raw images were analysed using the GenePix Pro 4.0 software (Axon). Duplicate hybridisations were performed for each sample to verify the reproducibility of the data except for one case (PC5). The correlation coefficients were calculated on the normalised tumour channel and observed to range from 0.63 to 0.90 (data not shown).

### Statistical and data analysis

The CGH arrays were read with the UCSF ‘SPOT’ software to produce raw text files (Jain *et al*, 2002). These files were read into R and normalised (using the loess intensity-dependent method), using the ‘limma’ package (Smyth, 2005; The R Development Core Team, 2006); background correction was omitted in this case, as visual inspection showed it increased the scatter in both the MA and chromosomal-location plots, and the correlation statistics were worse with background subtraction, indicative of low levels of background on the slides being misestimated. The log_2_-transformed normalised data were then pre-processed to average any on-slide replicates using the ‘snapCGH’ package from Bioconductor and segmented into local regions of constant copy number by circular binary segmentation (Gentleman *et al*, 2004; Olshen *et al*, 2004). The sample levels (two replicates for each tumour per clone) were summarised by means to give tumour-level data (one measurement per clone for each tumour). These data were then used in a linear model that estimated the fold change across the tumours, along with a *P*-value that the average log_2_-fold change between the tumour channel and the normal channel was non-zero (for both the normalised data and the locally smoothed data – data not shown for the latter). Clones that had an uncorrected *P*-value below 0.001 were considered to be significant candidates.

### Two-colour FISH

Four human BAC clones (RP11-403N12, RP11-232C20, RP11-8H2 and RP11-350K6) were purchased from BACPAC Resources (Oakland, CA, USA) and these DNAs were labelled with Cy3-dCTP using BioPrime Array CGH Genomic Labeling System (Invitrogen). Centromeric probes for chromosomes 7 and 18 (CEP7 and 18), labelled with SpectrumGreen, were purchased from Vysis (Downers Grove, IL, USA). The specificity of all the probes was confirmed by hybridisation onto Normal Male Metaphase (Vysis).

Two-colour FISH was performed as described previously (Lu *et al*, 1999). DNA copy number was evaluated for each probe by counting spots in at least 100 nuclei. A ratio was calculated between the average copy number of the BAC probes and of corresponding centromeric probes. Based on hybridisation in 10 normal pancreatic tissues (acinar and ductal cells), the threshold of gain and loss was defined as the ratios of >1.16 (+2 standard deviation (s.d.)) and <0.87 (−2 s.d.), respectively (data not shown). Our approach was to use normal samples to estimate overall noise levels: choosing the threshold on the tumour samples corresponding to a ±2 s.d. of the normal samples indicates a roughly 5% false-positive rate if the tumour samples were commensurate with diploidy.

### *In situ* RNA hybridisation for *SEC11L3*

The *SEC11L3* probe was amplified by PCR from OriGene clone TC123085 (OriGene Technologies, Inc., Rockville, MD, USA) that encodes full-length cDNA of *SEC11L3*. The primers used to amplify a 216-bp *SEC11L3* product are as follows: forward 5′-TTGGATATCTTCGGGGACCT-3′ and reverse 5′-GTCTTCCCGGAAATTTGTGA-3′. The PCR product was cloned into the pCR4-TOPO vector using the TOPO cloning kit (Invitrogen) to create pCR4-*SEC11L3*-ISH. Positive clones were verified by sequence analysis. The pCR4-*SEC11L3*-ISH plasmid was linearised with *Pst*I for the sense probe and *Not*I for the antisense probe. After removing restriction endonucleases, riboprobes were synthesised from 1 *μ*g of template DNA and digoxigenin (DIG) were labelled using a DIG RNA labeling kit (Roche Diagnostics GmbH, Mannheim, Germany). T3 and T7 polymerases were used to synthesise antisense probes and sense probes, respectively. DIG incorporation of riboprobes was verified by DOT blot with anti-digoxigenin-AP Fab fragments (Roche). Antisense and sense riboprobes for *SEC11L3* were hybridised to 19 tissue sections using the Ventana Discovery System with Ventana Ribomap and Bluemap kits. Expression of *SEC11L3* mRNA in cancer cells was compared to that of non-neoplastic epithelial cells (ductal, acinar, intestinal and hepatic cells) on the identical specimen and judged using a 0–2 score (0=no staining, 1=weak intensity, 2=intensity comparable to non-neoplastic counterparts).

### Reverse transcription–PCR for *SEC11L3*

cDNAs were synthesised from 1 *μ*g of total RNA using an oligo dT primer and the Multiscribe reverse transcription kit (Applied Biosystems, Warrington, Cheshire, UK) as instructed by the manufacturer. Reverse transcription was followed by 30 PCR cycles (1 min of denaturation at 94°C, 1 min of annealing at 55°C and 1 min of extension at 72°C). Primers for *SEC11L3* are the same as those designed in ISH. Primers for 18S ribosomal RNA, which was used as an endogenous control for normalisation, are as follows: forward 5′-CGCCGCTAGAGGTGAAATTC-3′ and reverse 5′-CATTCTTGGCAAATGCTTTCG-3′. Amplified products were separated on 1% agarose gels and visualised with ethidium bromide.

## RESULTS

### Comparison of array CGH profiles in microdissected tissues and cell lines

**Figure 1:**
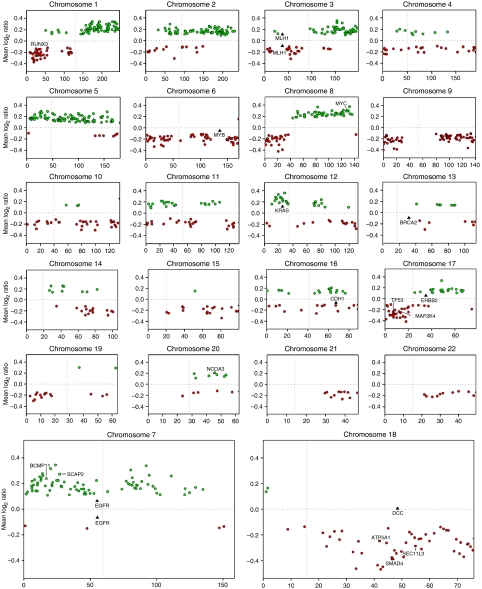
Summary of overall genome-wide alterations in a total of 23 microdissected PDAC tissues. Genetic gains are shown as green dots and losses as red dots (Y axis) at each clone position along the chromosome (X axis). Several representative clones with no genetic changes are depicted as black dots. Square-shaped dots indicate the clones validated by FISH, whereas triangle dots indicate previously identified genes in PDAC. Vertical dotted lines represent chromosome centromeres.

A total of 23 microdissected PDAC tissues were analysed by array CGH. Applying highly stringent statistical conditions (*P*<0.001), we could identify the regions of nonrandom genomic changes in PDAC. Figure 1 shows overall copy number changes for each chromosome and the entire data set of all clones is available in Supplementary Table 1 (the raw data set for all the experiments is shown in Supplementary Table 2). A total of 1015 clones met the statistical criterion; 17% of clones (508 clones including 698 candidate genes) showed genetic gain and 17% (507 clones including 1254 genes) showed loss. The array CGH profiles were compared to the previous reports in which PDAC-derived cell lines were analysed (Aguirre *et al*, 2004; Bashyam *et al*, 2005; Gysin *et al*, 2005; Nowak *et al*, 2005). Although they displayed similar spectrum patterns of genetic alterations overall, we found that there were apparently different breakpoints in our profiles. Our data showed several segmented gains on chromosome 2, which have rarely been observed in cell lines. In contrast, losses of 4q and 13q and gains of 11q and 20q in cell lines were not as frequent as in our microdissected samples. Next, we focused on individual clones harbouring the genes that are known to be dysregulated in cell lines. Increased copy numbers were detected in the regions including *MYC* (8q24.21) and *NCOA3/AIB1* (20q13.12), while genetic losses were observed in the regions containing *SMAD4* (18q21.1), *TP53* (17p13.1), *MAP2K4* (17p11.2) and *RUNX3* (1p36.11). However, using the rigorous statistical conditions employed, we identified neither genetic gains of *KRAS* (12p12.1), *MYB* (6q23.3), *EGFR* (7p11.2) and *ERBB2* (17q12) nor losses of *MLH1* (3p22.2), *BRCA2* (13q13.1) and *CDH1* (16q22.1). (All the genes cited here are depicted in Figure 1.)

### Contiguous regions of nonrandom copy number changes

In addition to numerous localised alterations, we detected a total of 41 contiguous regions (>3.0 Mb) of nonrandom genomic changes (Table 2). For instance, increased copy number was detected in the 26.0 Mb region of 7p22.2–p15.1 that contains 48 known or hypothetical protein-coding genes. We also defined the regions of genetic gains on 1q, 3q, 5p, 5q, 8q and 12p, which may represent loci for candidate oncogenes in PDAC. The largest region of copy number loss was from 17p13.3 to 17p12 (13.6 Mb), which covers a total of 53 candidate genes including *TP53* (17p13.1) as well as *MAP2K4* (17p11.2). We delineated three contiguous regions of genomic loss on 18q, which is known to be a site of frequent deletions in PDAC, 18q21.2–q22.1 (12.0 Mb), 18q22.3–q23 (7.1 Mb) and 18q12.3–q21.2 (6.9 Mb). The region of 18q21.2–q22.1 harbours 16 candidate genes in addition to *SMAD4* (18q21.1) that is one of the most recurrently inactivated tumour suppressor genes in PDAC. The region of 18q12.3–q21.2 contains a total of 23 putative target genes, whereas seven genes are included in the 7.1 Mb region of 18q22.3–q23. Interestingly, the clone encompassing *DCC* (18q21.2) has shown an apparent discontinuity between two broad regions of genetic loss (18q12.3–q21.2 and 18q21.2–q22.1) in our 23 microdissected PDAC sample set (Figure 1).

### Verification of genetic changes by two-colour FISH

**Figure 2:**
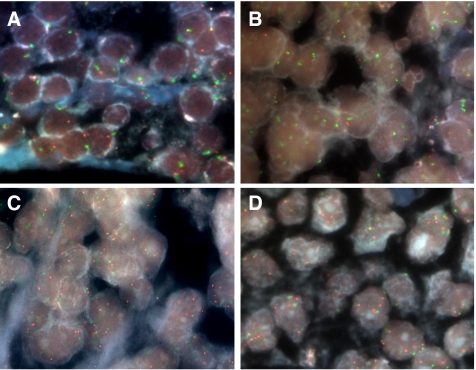
Four representative images in FISH analysis. Target BAC DNA probes were labelled with Cy3-dCTP (red), while centromeric probes were labelled with SpectrumGreen. DNA copy number was evaluated for each probe by counting spots in at least 100 nuclei. (**A**) No copy number change of *BCMP11* in PC40. (**B**) Genetic loss of *SEC11L3* in PC48. (**C**) Genetic gains of *BCMP11* in PC44 and (**D**) of *SCAP2* in PC49.

To investigate prospectively whether the identified genetic abnormalities are prevalent in PDAC, interphase FISH analysis was performed using an independent sample set (Figure 2). Previous cytogenetic studies have shown that chromosome arms 7p and 18q may include oncogenes and tumour suppressor genes that play a critical role in pancreatic carcinogenesis (Griffin *et al*, 1995; Hahn *et al*, 1995; Fukushige *et al*, 1997, 1998; Mahlamaki *et al*, 1997; Schleger *et al*, 2000; Harada *et al*, 2002a; Iacobuzio-Donahue *et al*, 2004). Therefore, we prioritised three regions (7p22.3–p15.1, 18q12.3–q21.2 and 18q21.2–q22.1) of nonrandom copy number changes detected in array CGH and a subset of four BAC clones (RP11-403N12 at 7p21.1, RP11-232C20 at 7p15.2, RP11-8H2 at 18q21.1 and RP11-350K6 at 18q21.32) were selected from those regions (Table 3). As shown in Table 4, deletion in the locus encompassing *SEC11L3* (18q21.32) was observed to be the most recurrent alteration (13 out of 19 samples; 68%) (Figure 2B). The region 18q21.1 defined by RP11-8H2 was deleted in 11 out of 19 cases (58%) and contains three known candidate genes: *ATP5A1*, *PSTPIP2* and *CCDC5*. RP11-403N12 including *BCMP11* (7p21.1) showed an increased copy number in 10 out of 19 cases (53%) (Figure 2C), whereas gain of the region at RP11-232C20 containing *SCAP2* (7p15.2) was demonstrated in seven out of 19 cases (37%) (Figure 2D).

### *SEC11L3* mRNA downregulation detected by ISH and RT–PCR

**Figure 3:**
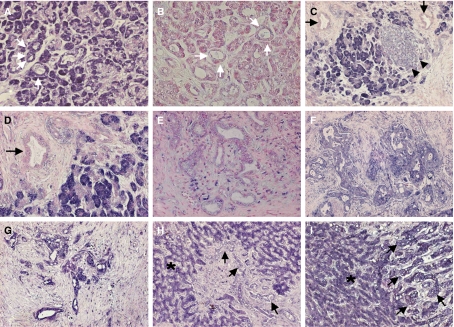
*SEC11L3* mRNA expression in non-neoplastic epithelial cells and PDAC cells, as determined by ISH. (**A**) Strong expression (score 2) in both ductal (*white arrows*) and acinar cells of non-neoplastic pancreas. (**B**) ISH conducted with a sense *SEC11L3* riboprobe, used as a negative control. (**C**–**E**). No expression (score 0) in PDAC cells (*black arrows*), but weak expression (score 1) in non-neoplastic islet cells (*black arrow heads*) (PC41 and 47) (**F**). Weak expression (score 1) of PDAC cells in PC43 and (**G**) strong intensity of expression (score 2) of PDAC cells in PC44. (**H**) Lower level of expression (score 1; PC53) and (**I**) similar intensity of expression (score 2; PC55) in metastatic PDAC cells (*black arrows*) compared to non-neoplastic hepatic cells (*asterisk*).

Subsequently, the *SEC11L3* mRNA level was evaluated by ISH (Figure 3). Firstly, we tested several different types of normal epithelial cells from the pancreas, intestine and liver. Normal pancreatic tissues showed strong mRNA expression of *SEC11L3* in both ductal and acinar cells (score 2), whereas there was weak expression in islet cells (score 1). Strong expression was also observed in normal intestinal and hepatic cells (score 2). Of 19 PDAC cases, *SEC11L3* mRNA was downregulated (score 0–1) in 11 cases (58%), whereas it was unchanged (score 2) in eight cases (42%) (Table 4). All 11 cases with downregulated *SEC11L3* demonstrated genetic losses by FISH. Despite decreased copy numbers, *SEC11L3* was expressed in two samples (PC46 and PC51). A significant correlation between deletion and expression levels was found (*P*=0.001, Fisher's exact test), indicating that the mRNA level of this gene was highly dependent on its DNA copy number.

**Figure 4:**
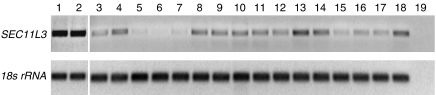
mRNA expression of *SEC11L3* in normal pancreas and PDAC tissues, as determined by RT-PCR. 18S ribosomal RNA was used as an internal standard. Samples were run in the following order: lane 1–2, normal pancreas; lane 3–18, PDACs; lane 19, negative control. *SEC11L3* expression was found to be present in normal pancreas, while it was decreased in 16 out of 16 PDAC tissues (100%).

RT–PCR was performed further to confirm downregulated mRNA of *SEC11L3* in PDAC. Figure 4 shows *SEC11L3* expression in normal pancreas and PDAC tissue samples. Compared to two normal pancreas tissues, *SEC11L3* was found to be downregulated in 16 out of 16 PDAC samples (100%). In particular, the *SEC11L3* transcript was virtually almost absent in four PDAC tissues (25%; lane 5, 6, 7 and 15).

## DISCUSSION

It is well known that a strong desmoplastic reaction is a typical feature of PDAC tissues. A dense stromal component, which occupies larger parts of the tumour mass, significantly reduces the sensitivity and specificity of cytogenetic analysis. Tissue microdissection is laborious, but the practical method available to enrich the tumour cell population in clinical specimens. In the present study, we first identified genomic abnormalities that represent the characteristics of tumour cells *in vivo* by combining CGH arrays with tissue microdissection. This approach led to more precise definition of chromosomal breakpoints in a panel of 23 PDAC tissue samples. To identify nonrandom genomic changes in PDAC, we applied a *P*-value rather than a fixed cutoff value because we found that concomitant lack of power in dichotomising the data at an early stage in the analysis provided poor resolution with which to distinguish between clones. Taking a statistical threshold approach allows us to take account of different clones’ differing variance across samples that a fixed fold-change approach does not.

We compared the CGH profiles to the previously published cell line data (Karhu *et al*, 2006). Despite overall similar spectrum patterns, there were clear differences between both profiles. It is important to take into account that the resolution of CGH arrays used and the type of statistical analysis employed vary widely between the reports. However, our results indicated that some recurrent genetic alterations, such as losses of 4q and 13q and gains of 11q and 20q, seem to be relatively unique to cell lines, implying that these genetic changes may have been artificially acquired through the establishment of cell lines or in the course of culturing. In addition, our data did not demonstrate significant copy number changes of some known genes, such as *KRAS*, *ERBB2*, *MLH1* and *CDH1*. This is probably due to the fact that intragenic mutation or promoter methylation is more likely to occur in these genes (Lemoine *et al*, 1992; Scarpa *et al*, 1994; Rozenblum *et al*, 1997; Ueki *et al*, 2000). Alternatively, this discrepancy could be explained by the intratumoral heterogeneity that is characteristically observed in PDAC cells *in vitro* as well as *in vivo*, or may reflect the differences of the geographic origin of the tumours used in this study (a total of 42 Japanese samples were analysed by array CGH, FISH and ISH) (Scarpa *et al*, 1994; Gorunova *et al*, 1998; Harada *et al*, 2002b). Similarly, deletion of the DCC gene was not recurrent in our sample set. However, a larger scale of study using much higher-resolution genome-wide microarrays (tiling-path CGH arrays or single nucleotide polymorphism arrays) is required to conclude whether this genetic alteration is critically involved in the pathogenesis of PDAC.

As our array CGH profiles revealed that the regions of 7p22.2–p15.1, 18q12.3–q21.2 and 18q21.2–q22.1 are nonrandomly altered, four clones from those regions were validated by FISH experiments. The results showed that genetic alterations for these clones were observed in 37–68% of tumours in a separate sample set, supporting the validity of our CGH results. Moreover, copy number changes of those four clones were detected in 18 out of 19 PDAC cases (95%), implying the potential clinical applicability. Among the candidate genes verified by FISH, *BCMP11* is newly detected in PDAC, although Fletcher *et al* (2003) demonstrated that mRNA and protein of this gene were overexpressed in oestrogen receptor-positive breast cancer. This gene lies adjacent to the *AGR2* gene (7p21.1) and both genes are classified as members of the same family (AGR family) due to highly similar (approximately 70%) protein sequences. Interestingly, several gene expression analyses have shown that *AGR2* is upregulated in the majority of PDACs as well as pancreatic intraepithelial neoplasia (PanIN) lesions (Crnogorac-Jurcevic *et al*, 2003; Iacobuzio-Donahue *et al*, 2003a; Iacobuzio-Donahue *et al*, 2003b; Missiaglia *et al*, 2004; Buchholz *et al*, 2005). Taken together, *BCMP11* is also likely to be involved in the development of PDAC. On the other hand, *SCAP2* was first described in a recently published report, showing that mRNA of this gene is frequently overexpressed in PanIN lesions (Buchholz *et al*, 2005). The protein encoded by this gene belongs to the src family of kinases. Takahashi *et al* (2003) demonstrated that *SCAP2* functions as a downstream target of c-Src under various stress conditions (UV light, tumour necrosis factor-*α* and osmotic stress). Therefore, *SCAP2* also seems to work as a cell-signalling molecule in cancer cells. However, the biological function and putative role of these two genes have not been investigated in cancer.

Remarkably, *SEC11L3* was deleted in approximately 70% of tumours and its expression level was significantly correlated with its DNA copy number. Despite the high frequency of this genetic abnormality, this gene has not been described in any type of cancer. Previous cytogenetic analyses have revealed a frequent deletion of 18q in PDAC, but neither the chromosomal breakpoints nor the candidate genes included could be clearly identified due to technical limitations of the technology employed (Griffin *et al*, 1995; Hahn *et al*, 1995; Fukushige *et al*, 1997, 1998; Mahlamaki *et al*, 1997; Hoglund *et al*, 1998; Schleger *et al*, 2000; Harada *et al*, 2002a; Iacobuzio-Donahue *et al*, 2004). *SMAD4* (18q21.1) has been reported to be deleted or inactivated in about 50% of PDACs and, therefore, it is considered to be one of the most likely candidate tumour suppressor genes at this locus (Hahn *et al*, 1996; Rozenblum *et al*, 1997). However, we propose that *SEC11L3* (18q21.32) could be an equally promising candidate gene on 18q because the significance of genetic loss of this gene is comparable to that of the *SMAD4* gene. In addition, both ISH and RT–PCR independently confirmed that *SEC11L3* mRNA is downregulated in PDAC tissues at a high frequency (53% and 100%, respectively), suggesting that dysregulation of this gene is likely to be associated with the development of PDAC. Little is known about the biological role of this gene, although it belongs to the peptidase S26B family and functions as part of the signal peptidase complex.

In summary, we could successfully identify genetic alterations that reflect the intrinsic characteristics of PDAC cells *in vivo* by combining array CGH with tissue microdissection. These results provided a valid data set to search for novel candidate genes involved in pancreatic carcinogenesis. The specificity of our array CGH results was confirmed by interphase FISH in an independent sample set. Among the identified candidates, we are particularly interested in the *SEC11L3* gene that is located on 18q21.32. FISH and ISH analyses for this gene demonstrated a significant correlation between genetic deletion and the corresponding mRNA downregulation, raising the possibility that the *SEC11L3* gene may play a putative role as a tumour suppressor. For these reasons, we propose that *SEC11L3* should be considered as a potential marker gene for the molecular diagnosis of PDAC and a possible candidate target for therapeutic intervention.

## Figures and Tables

**Table 1:** The clinicopathological data of PDAC tissue samples

**Sample**	**Age**	**Sex**	**Location^a^**	**Histology^b^**	**T**	**N**	**M**	**Stage**
*(A) Microdissected fresh-frozen tissue samples used for array CGH (n=23)*								
PC1	66	F	P(Ph)	mod	3	0	0	II
PC2	66	F	P(Pb)	well	3	1a	0	III
PC3	66	M	P(Ph)	mod	3	1b	0	III
PC4	59	F	P(Ph)	mod	3	1b	0	III
PC5	64	M	P(Ph)	mod	3	1b	0	III
PC6	47	M	P(Ph)	mod	4	1b	0	IVa
PC7	66	M	P(Ph)	mod	4	1b	0	IVa
PC8	69	F	P(Ph)	poor	4	1b	0	IVa
PC9	73	F	P(Ph)	mod	4	1b	0	IVa
PC10	56	M	P(Pt)	mod	4	1b	1	IVb
PC11	76	F	P(Ph)	mod	4	1b	1	IVb
PC12	63	F	P(Pb)	mod	4	1b	1	IVb
PC13	78	F	P(Pt)	mod	4	1b	1	IVb
PC14	68	F	P(Ph)	mod	4	1b	1	IVb
PC15	65	M	P(Pb)	mod	4	1b	1	IVb
PC16	65	M	P(Ph)	mod	4	1b	1	IVb
PC17	60	M	P(Ph)	mod	4	1b	1	IVb
PC18	76	M	P(Ph)	mod	4	1b	1	IVb
PC19	54	F	P(Pb)	poor	4	1b	1	IVb
PC20	72	F	P(Pt)	mod	4	1b	1	IVb
PC34	60	M	LM	mod	4	1b	1	IVb
PC35	75	M	LM	mod	4	1b	1	IVb
PC36	67	F	LM	mod	4	1b	1	IVb
								
*(B) Formalin-fixed, paraffin-embedded tissue sections used for FISH and ISH (n=19)*								
PC37	74	M	P(Ph)	mod	2	0	0	I
PC38	72	M	P(Pt)	poor	3	0	0	II
PC39	70	M	P(Ph)	mod	3	1	0	III
PC40	58	M	P(Ph)	mod	3	1	0	III
PC41	69	F	P(Pb)	well	3	1	0	III
PC42	51	M	P(Ph)	poor	3	1	0	III
PC43	73	M	P(Ph)	mod	3	1	0	III
PC44	59	M	P(Pb)	mod	3	1	0	III
PC45	53	F	P(Ph)	mod	4	1	0	IVa
PC46	53	M	P(Pb)	mod	4	1	0	IVa
PC47	56	M	P(Ph)	well	4	1	0	IVa
PC48	57	F	P(Ph)	mod	3	1	1	IVb
PC49	65	F	P(Ph)	mod	3	1	1	IVb
PC50	59	M	P(Ph)	mod	3	1	1	IVb
PC51	61	M	P(Ph)	mod	4	1	1	IVb
PC52	74	M	P(Pb)	poor	4	1	1	IVb
PC53	57	F	LM	mod	3	1	1	IVb
PC54	61	M	LM	mod	4	1	1	IVb
PC55	74	M	LM	poor	4	1	1	IVb

a P=primary lesion; Ph=head; Pb=body; Pt=tail of the pancreas; LM=liver metastatic lesion.

b mod=moderately; poor=poorly differentiated tubular adenocarcinoma.

PDAC=pancreatic ductal adenocarcinoma; FISH=fluorescence *in situ* hybridisation; ISH=*in situ* RNA hybridisation.

**Table 2:** Contiguous regions (>3.0 Mb) of chromosomal changes in a total of 23 microdissected PDAC tissues

**Locus**	**Start (bp)**	**End (bp)**	**Size (Mb)**	**Mean log2**	**Locus**	**Start (bp)**	**End (bp)**	**Size (Mb)**	**Mean log2**
1q24.1–q25.1	163 809 021	173 127 283	9.3	0.235	1p35.1–p34.3	34 152 076	38 379 441	4.2	−0.232
1q25.2–q25.3	177 942 453	181 046 133	3.0	0.208	4p16.2–p16.1	5 094 062	8 313 477	3.2	−0.216
1q31.1–q31.3	187 406 225	1 957 10 013	8.3	0.255	6p21.32–p21.31	32 031 967	34 013 145	4.0	−0.219
1q41	215 371 867	219 919 554	4.5	0.242	6q21	108 154 127	112 486 834	4.3	−0.181
1q42.2–q43	231 700 764	241 495 322	9.8	0.253	6q25.2–q25.3	155 289 734	158 440 778	3.2	−0.204
2p16.1–p14	60 927 185	64 676 357	4.8	0.171	8p22–p21.3	17 784 184	21 872 595	4.1	−0.264
2q22.2–q22.3	143 499 638	146 775 971	3.3	0.168	9p24.3–p24.1	190	6 659 690	6.7	−0.228
2q32.1	185 140 061	188 357 186	3.2	0.161	9p22.1–p21.3	19 310 506	23 557 472	4.2	−0.256
2q32.3	192 914 919	196 752 218	3.8	0.146	9q22.31–q22.33	94 281 893	100 889 311	6.6	−0.171
3q26.1	163 532 324	167 501 265	4.0	0.237	9q33.3–q34.11	126 127 921	129 390 787	3.3	−0.216
5p15.31–p15.2	7 449 057	10 495 937	3.0	0.165	9q34.13–q34.3	133 982 008	137 333 442	3.4	−0.226
5p14.3–p14.1	20 429 524	28 918 008	8.5	0.232	17p13.3–p12	800 495	14 360 892	13.6	−0.252
5q11.1–q11.2	50 061 482	54 725 678	4.7	0.174	18q12.3–q21.2	41 216 566	48 119 508	6.9	−0.350
5q12.2–q13.1	63 325 516	66 888 015	3.6	0.149	18q21.2–q22.1	49 795 841	61 752 947	12.0	−0.252
5q14.1–q14.3	80 075 105	86 472 400	6.4	0.168	18q22.3–q23	68 809 458	75 940 259	7.1	−0.294
5q14.3	87 676 680	90 964 362	3.3	0.170	21q21.3–q22.11	29 627 040	34 602 938	5.0	−0.182
7p22.2–p15.1	2 607 390	28 603 446	26.0	0.207	22q11.22–q12.1	21 400 636	25 896 652	4.5	−0.205
7p14.1	38 185 228	41 345 287	3.2	0.170	22q12.2-q12.3	28 379 384	31 627 100	3.2	−0.180
7q21.11	79 539 131	83 414 012	3.9	0.219					
8q21.11–q21.13	77 234 275	81 648 851	4.4	0.225					
8q21.13–q21.3	82 640 038	89 400 934	6.8	0.271					
8q24.11–q24.13	118 297 084	123 697 997	5.4	0.254					
12p12.3–p12.1	19 403 674	23 784 534	4.4	0.268					

PDAC=pancreatic ductal adenocarcinoma.

**Table 3:** Contiguous regions of nonrandom copy number changes on 7p and 18q

**BAC**					**Region**	**Genes included^a^**		**Overall results**		
**Clone ID^b^**	**Cytoband**	**Start (bp)**	**End (bp)**	**Size (bp)**	**Size (Mb)^c^**	**No**	**Plausible candidates**	**Mean log2**	**Fold changes**	***P*-value**
RP11-106E3	7p22.2	2 607 390	2 657 138	49 749	26.0	2	*IQCE*	0.134	1.098	0.000242
RP11-172O13	7p22.1	5 704 716	5 847 079	142 364		1	*TRIAD3*	0.182	1.135	5.25E-06
RP1-42M2	7p22.1	59 69 700	6 049 710	80 011		7	*PMS2, EIF2AK1*	0.165	1.121	0.000272
RP4-810E6	7p22.1	6 049 510	6 202 437	152 928		6	*PSCD3, EIF2AK1*	0.220	1.164	1.23E-06
RP11-425P5	7p22.1	6 233 987	6 446 613	212 627		4	*RAC1, PSCD3*	0.144	1.105	0.000254
RP4-733B9	7p22.1-p21.3	7 176 436	7 292 189	115 754		1	*C1GALT1*	0.189	1.140	4.92E-07
RP11-505D17	7p21.3	7 947 759	8 125 919	178 161		2	*GLCCI1, ICA1*	0.231	1.174	1.07E-08
RP11-304A10	7p21.3	8 950 512	9 040 173	89 662		0		0.144	1.105	0.000274
RP5-959C21	7p21.3	9 924 891	10 064 840	139 950		0		0.143	1.104	6.08E-06
RP11-352E12	7p21.3	10 064 640	10 151 963	87 324		0		0.190	1.141	2.08E-06
RP11-392P1	7p21.3	10 372 373	10 429 780	57 408		0		0.207	1.155	3.66E-08
RP11-502P9	7p21.3	11 782 368	11 938 707	156 340		0		0.254	1.192	5.32E-09
RP5-1100A7	7p21.3	12 725 574	12 827 381	101 808		0		0.226	1.170	9.02E-09
RP11-195L14	7p21.3	12 951 352	13 077 542	126 191		0		0.242	1.183	1.45E-08
RP4-685A2	7p21.2	13 887 063	13 996 422	109 360		1	*ETV1*	0.278	1.213	5.64E-10
RP11-512E16	7p21.2	14 128 072	14 279 388	151 317		1	*DGKB*	0.262	1.199	1.75E-09
RP11-196O16	7p21.1	16 023 294	16 205 465	182 172		2		0.182	1.135	8.23E-07
**RP11-403N12**	**7p21.1**	**16 865 247**	**17 055 918**	**190 672**		**1**	***BCMP11***	**0.239**	**1.181**	**2.76E-08**
RP11-323K15	7p21.1	17 781 327	17 929 240	147 914		1	*SNX13*	0.109	1.079	9.16E-05
RP11-71F18	7p21.1-p15.3	19 406 242	19 580 569	174 328		0		0.147	1.107	8.00E-06
RP11-486P11	7p15.3	20 042 179	20 150 597	108 419		1	*7A5*	0.317	1.246	1.30E-11
RP4-701O19	7p15.3	20 884 182	20 950 414	66 233		0		0.190	1.140	4.72E-05
RP11-211J15	7p15.3	21 173 901	21 257 598	83 698		2		0.183	1.135	2.43E-07
RP11-445O1	7p15.3	21 588 234	21 669 042	80 809		1	*DNAH11*	0.210	1.157	9.97E-08
RP11-451F11	7p15.3	23 714 553	23 804 532	89 980		1	*STK31*	0.346	1.271	1.43E-11
RP11-343P21	7p15.3	24 511 504	24 521 807	10 304		0		0.226	1.169	3.17E-08
RP11-99O17	7p15.2	25 824 267	25 925 677	101 411		0		0.225	1.169	2.79E-07
**RP11-232C20**	**7p15.2**	**26 765 660**	**26 911 371**	**145 712**		**1**	***SCAP2***	**0.275**	**1.210**	**1.80E-10**
RP4-781A18	7p15.2-p15.1	27 976 171	28 166 812	190 642		2	*tcag7.981*	0.181	1.133	1.83E-06
RP4-596O9	7p15.1	28 459 769	28 603 446	143 678		1	*CREB5*	0.175	1.129	8.62E-06
										
RP11-463D17	18q12.3	41 216 566	41 408 713	192 148	6.9	0		−0.439	−1.356	1.00E-11
**RP11-8H2**	**18q21.1**	**41 851 567**	**41 984 947**	**133 381**		**5**	***ATP5A1, CCDC5, PSTPIP2***	**−0.249**	**−1.189**	**1.50E-07**
RP11-313C14	18q21.1	423 50 383	42 350 954	572		1	*LOXHD1*	−0.471	−1.386	5.75E-13
RP11-71F23	18q21.1	43 069 131	43 274 185	205 055		0		−0.451	−1.367	2.87E-11
RP11-46D1	18q21.1	44 393 176	44 552 924	159 749		1	*KIAA0427*	−0.264	−1.201	1.85E-05
RP11-141E12	18q21.1	45 025 819	4 5187 979	162 161		1	*DYM*	−0.150	−1.109	0.000150
RP11-419L16	18q21.1-q21.2	46 310 160	46 473 840	163 681		1	*MAPK4*	−0.378	−1.300	1.02E-06
RP11-76E22	18q21.2	46 462 730	46 633 371	170 642		2	*MRO*	−0.393	−1.313	3.94E-10
RP11-729G3	18q21.2	46 732 284	46 888 494	156 211		4	*SMAD4, ELAC1*	−0.389	−1.309	2.67E-08
RP11-1E21	18q21.2	47 274 828	47 443 303	168 476		2		−0.322	−1.250	2.22E-08
RP11-25O3	18q21.2	47 958 320	48 119 508	161 189		0		−0.346	−1.271	1.64E-06
										
RP11-116K4	18q21.2	49 795 841	49 971 830	175 990	12.0	1	*MBD2*	−0.331	−1.258	6.44E-08
RP11-99A1	18q21.2	50 563 151	50 702 093	138 943		1	*RAB27B*	−0.374	−1.296	4.86E-08
RP11-397A16	18q21.2	51 445 553	51 648 118	202 566		1		−0.263	−1.200	1.58E-07
RP11-383D22	18q21.31	52 656 265	52 867 730	211 466		2	*WDR7*	−0.179	−1.132	0.000196
RP11-35G9	18q21.31	53 447 744	53 561 700	113 957		4	*ATP8B1*	−0.195	−1.145	1.99E-06
RP11-61J14	18q21.32	54 567 090	54 747 580	180 491		6	*ZNF532, MALT1*	−0.223	−1.167	3.87E-07
**RP11-350K6**	**18q21.32**	**54 867 252**	**55 027 999**	**160 748**		**1**	***SEC11L3***	−**0.289**	**−1.222**	**5.58E-09**
RP11-396N11	18q21.32	56 063 594	56 151 592	87 999		0		−0.233	−1.176	7.74E-08
RP11-520K18	18q21.32	56 874 822	57 034w619	159 798		0		−0.230	−1.173	1.22E-06
RP11-13L22	18q21.33	58 408 978	58 578 530	169 553		3	*PHLPP*	−0.320	−1.248	3.63E-11
RP11-215A20	18q21.33	58 572 412	58 756 503	184 092		2	*PHLPP*	−0.198	−1.147	1.24E-05
RP11-233O10	18q22.1	59 886 252	59 971 318	85 067		1	*C18orf20*	−0.250	−1.189	2.21E-10
RP11-389J22	18q22.1	61 594 898	61 752 947	158 050		1	*CDH7*	−0.189	−1.140	8.76E-07
										
RP11-169F17	18q22.3	68 809 458	69 000 813	191 356	7.1	0		−0.329	−1.256	4.02E-10
RP11-25L3	18q22.3	69 588 036	69 755 177	167 142		0		−0.236	−1.177	8.63E-08
RP11-556L15	18q22.3	70 753 437	70 931 323	177 887		1	*ZNF407*	−0.348	−1.272	3.55E-09
RP11-396D4	18q22.3–q23	71 168 342	71 337 306	168 965		1		−0.261	−1.198	1.51E-07
RP11-234N1	18q23	72 266 630	72 448 118	181 489		2	*ZNF516*	−0.373	−1.295	3.41E-11
RP11-118I2	18q23	73 613 846	73 764 173	150 328		0		−0.275	−1.210	1.20E-06
RP11-16L7	18q23	73 908 671	74 017 409	108 739		0		−0.271	−1.206	2.94E-12
RP11-563B11	18q23	74 707 626	74 870 951	163 326		1	*SALL3*	−0.294	−1.226	3.81E-07
RP11-154H12	18q23	75 586 355	75 701 258	114 904		2	*CTDP1*	−0.321	−1.249	1.20E-08
CTC-964M9	18q23	75 939 424	75 940 259	836		0		−0.230	−1.173	9.86E-06

a The four clones that were used in FISH analysis are outlined in bold.

b The size of the contiguous region.

c The number of genes included in each clone and examples of candidate genes contained within each clone. All the details are shown in Supplementary Table 1.

**Table 4:** The results of FISH and ISH analyses^a^

**Clone ID/candidate**	**RP11-403N12**/***BCMP11***	**RP11-232C20**/***SCAP2***	**RP11-8H2**/***ATP5A1***	**RP11-350K6**/***SEC11L3***	
**Analysis**	**FISH**	**FISH**	**FISH**	**FISH**	**ISH**
PC37	1.05	0.99	**0.71**	**0.83**	**1**
PC38	1.02	1.12	0.94	0.94	2
PC39	0.98	1.09	1.05	**0.77**	**0**
PC40	1.01	**1.30**	0.95	**0.57**	**1**
PC41	1.13	0.93	**0.20**	**0.46**	**0**
PC42	**1.28**	**1.18**	**0.56**	0.91	2
PC43	**1.23**	0.94	0.91	**0.71**	**1**
PC44	**1.35**	**1.52**	**0.66**	0.94	2
PC45	1.01	1.01	**0.63**	**0.71**	**1**
PC46	1.06	1.13	0.94	**0.86**	2
PC47	0.98	1.03	**0.74**	**0.36**	**0**
PC48	**1.59**	0.94	0.90	**0.51**	**0**
PC49	**1.17**	**1.24**	**0.58**	1.04	2
PC50	1.01	1.03	0.94	**0.63**	**0**
PC51	**1.24**	**1.26**	**0.64**	**0.82**	2
PC52	**1.25**	0.97	**0.85**	1.11	2
PC53	**1.49**	1.14	**0.71**	**0.70**	**1**
PC54	**1.39**	**1.50**	**0.51**	**0.52**	**0**
PC55	**1.41**	**1.50**	0.95	1.08	2
					
Frequency (%)	10/19 (53%)	7/19 (37%)	11/19 (58%)	13/19 (68%)	11/19 (58%)

a Significant differences are outlined in bold.

FISH=fluorescence *in situ* hybridisation; ISH=*in situ* RNA hybridisation.
